# Supplementary material for: Gating mechanisms during actin filament elongation by formins
Source: eLife. 2018 Jul 23;7:e37342. doi: 10.7554/eLife.37342 (PMC6056239; doi:10.7554/eLife.37342)
Supplement: Supplementary file 1. — The columns show feature name and residues comprising corresponding feature. [file elife-37342-supp1.docx]

| **Feature name** | **Cdc12** | **Bni1** | **mDia1** |
| --- | --- | --- | --- |
| **Lasso** | 984–1029 | 1350–1393 | 754–796 |
| **Linker** | 1030–1047 | 1394–1415 | 797–829 |
| **Knob** | 1048–1147 | 1416–1521 | 830–913 |
| **KnA** | 1055–1073 | 1422–1440 | 836–853 |
| **KnB** | 1090–1111 | 1457–1479 | 868–887 |
| **Coiled-coil** | 1148–1188, 1272–1340 | 1522–1562, 1646–1714 | 914–953, 1037–1104 |
| **Post** | 1189–1271, 1341–1385 | 1563–1645, 1715–1760 | 954–1036, 1105–1150 |
