# Supplementary material for: Gating mechanisms during actin filament elongation by formins
Source: eLife. 2018 Jul 23;7:e37342. doi: 10.7554/eLife.37342 (PMC6056239; doi:10.7554/eLife.37342)
Supplement: Supplementary file 2. — TM-score is used to determine the similarity of protein structures. Based on statistics, TM-score between 0.0 and 0.17 indicates random structural similarity and TM-score between 0.5 and 1.00 indicates having about the same fold [43]. TM-scores were obtained through this website: https://zhanglab.ccmb.med.umich.edu/TM-align/ [file elife-37342-supp2.docx]

| Template Model Score (TM-score) | |
| --- | --- |
| **Bni1 based on Cdc12** | |
| FHL | 0.791 |
| FHT | 0.792 |
| **Cdc12 based on mDia1** | |
| FHL | 0.652 |
| FHT | 0.614 |
| **mDia1 based on Cdc12** | |
| FHL | 0.747 |
| FHT | 0.726 |
