# Supplementary material for: Gating mechanisms during actin filament elongation by formins
Source: eLife. 2018 Jul 23;7:e37342. doi: 10.7554/eLife.37342 (PMC6056239; doi:10.7554/eLife.37342)
Supplement: Supplementary file 3. — Pearson correlation coefficients for the number of contacts between the lasso, knob and post regions of the FH2 domains and actin subunits (A2 and A3) and the distributions of twist angles of A2-A3 (given in Figure 5—figure supplement 2 and Figure 5—figure supplement 1) during 350 ns all-atom MD simulations of five-mer filaments. [file elife-37342-supp3.docx]

| **Region**  **Formin** | **FHL knob – A2** | **FHT knob – A3** | **FHL lasso – A2** | **FHL post – A2** | **FHL post – A3** | **FHT post – A2** | **FHT post – A3** |
| --- | --- | --- | --- | --- | --- | --- | --- |
| **Cdc12** | 0.144 | 0.103 | 0.331 | 0.744 | 0.568 | 0.480 | 0.498 |
| **Bni1** | 0.022 | 0.044 | 0.002 | 0.09 | 0.084 | -0.076 | -0.066 |
| **mDia1** | 0.011 | -0.236 | 0.068 | 0.209 | 0.053 | 0.308 | -0.14 |
