# Supplementary material for: Gating mechanisms during actin filament elongation by formins
Source: eLife. 2018 Jul 23;7:e37342. doi: 10.7554/eLife.37342 (PMC6056239; doi:10.7554/eLife.37342)
Supplement: Supplementary file 4. — The average number of contacts (with standard deviations and t-statistics) between the lasso, knob and post regions of the FH2 domains and actin subunits (A2 and A3) from 350 ns of the all-atom MD simulations of five-mer filaments. [file elife-37342-supp4.docx]

| **Region**  **Formin** | **FHL knob – A2** | **FHT knob – A3** | **FHL lasso – A2** | **FHL post – A2** | **FHL post – A3** | **FHT post – A2** | **FHT post – A3** |
| --- | --- | --- | --- | --- | --- | --- | --- |
| **Cdc12** | 289±22 | 249±32 | 40±10 | 82±38 | 60±19 | 127±13 | 79±20 |
| **Bni1** | 253±23 | 269±33 | 45±11 | 112±16 | 63±15 | 93±21 | 72±9 |
| **mDia1** | 248±19 | 249±38 | 5±3 | 29±8 | 53±10 | 138±17 | 63±8 |
| **T-statistics** | | | | | | | |
| **Cdc12 – Bni1** | 6.5 | 2.5 | 1.7 | 4.2 | 0.7 | 7.9 | 2.0 |
| **Cdc12 – mDia1** | 8.3 | 0.03 | 20.0 | 7.9 | 1.9 | 3.0 | 4.6 |
| **Bni1 – mDia1** | 1.1 | 2.3 | 19.9 | 27.3 | 3.3 | 9.6 | 4.7 |
